# Retrospective checking of compliance with practice guidelines for acute stroke care: a novel experiment using openEHR’s Guideline Definition Language

**DOI:** 10.1186/1472-6947-14-39

**Published:** 2014-05-10

**Authors:** Nadim Anani, Rong Chen, Tiago Prazeres Moreira, Sabine Koch

**Affiliations:** 1 Health Informatics Centre, LIME, Karolinska Institutet, Tomtebodavägen 18, SE 17177 Stockholm, Sweden; 2 Cambio Healthcare Systems, Ringvägen 100, SE 11860 Stockholm, Sweden; 3 Department of Neurology, Karolinska Stroke Research Unit, Karolinska University Hospital-Solna, SE 17176 Stockholm, Sweden; 4 Department of Clinical Neuroscience, Stroke Research Group, Karolinska Institutet, SE 17177 Stockholm, Sweden

**Keywords:** Computer-assisted decision making, Practice guideline, Guideline adherence, Electronic health records, Semantics, openEHR, Artificial intelligence, Stroke

## Abstract

**Background:**

Providing scalable clinical decision support (CDS) across institutions that use different electronic health record (EHR) systems has been a challenge for medical informatics researchers. The lack of commonly shared EHR models and terminology bindings has been recognised as a major barrier to sharing CDS content among different organisations. The openEHR Guideline Definition Language (GDL) expresses CDS content based on openEHR archetypes and can support any clinical terminologies or natural languages. Our aim was to explore in an experimental setting the practicability of GDL and its underlying archetype formalism. A further aim was to report on the artefacts produced by this new technological approach in this particular experiment. We modelled and automatically executed compliance checking rules from clinical practice guidelines for acute stroke care.

**Methods:**

We extracted rules from the European clinical practice guidelines as well as from treatment contraindications for acute stroke care and represented them using GDL. Then we executed the rules retrospectively on 49 mock patient cases to check the cases’ compliance with the guidelines, and manually validated the execution results. We used openEHR archetypes, GDL rules, the openEHR reference information model, reference terminologies and the Data Archetype Definition Language. We utilised the open-sourced GDL Editor for authoring GDL rules, the international archetype repository for reusing archetypes, the open-sourced Ocean Archetype Editor for authoring or modifying archetypes and the CDS Workbench for executing GDL rules on patient data.

**Results:**

We successfully represented clinical rules about 14 out of 19 contraindications for thrombolysis and other aspects of acute stroke care with 80 GDL rules. These rules are based on 14 reused international archetypes (one of which was modified), 2 newly created archetypes and 51 terminology bindings (to three terminologies). Our manual compliance checks for 49 mock patients were a complete match versus the automated compliance results.

**Conclusions:**

Shareable guideline knowledge for use in automated retrospective checking of guideline compliance may be achievable using GDL. Whether the same GDL rules can be used for at-the-point-of-care CDS remains unknown.

## Background

The benefits of clinical practice guidelines (from now on referred to as ‘guidelines’) in the practice of evidence-based medicine have been known for a long time [1]. Guidelines may improve care by providing better clinical outcomes, ensuring patient safety, reducing costs and decreasing care variability [1]. This has led to a great interest in utilising guidelines for providing patient-specific recommendations at the point of clinical decision making and for checking compliance with guidelines retrospectively. The most effective way to do that seems to be the computerised execution of guidelines in order to provide clinical decision support (CDS) [1].

The main approach to achieving computerised execution of guidelines in medical informatics research has been the use of languages that can create computer-interpretable guidelines, i.e. guidelines that a computer can run automatically. These languages, sometimes known as guideline representation models, include PROforma [2], Asbru [3], Arden Syntax [4], GLIF [5], GUIDE [6], SAGE [7] and others. The Arden Syntax was a pioneering rule-based effort and is perhaps one of the best known guideline representation models, while also being famous for its ‘curly braces problem’: the lack of standardised patient data formats caused by the Arden Syntax’s local data definitions within Medical Logic Modules [8].

According to a review by Wang et al. all available guideline representation models support the two clinical tasks of actions and decisions, where actions can be any type of clinical intervention that changes the state of the patient or data collections about the patient, and decisions are choices made based on different alternatives [9]. They further establish that these languages usually try to explicitly model patient states based on actions and decisions. Another review by Isern and Moreno takes into account the tooling support provided for different guideline representation models, e.g. whether a graphical editor is provided to do the modelling and what sort of functionality the guideline execution engine provides in terms of coordinating scheduled plans and complex temporal conditions to the satisfaction of users [10].

Both reviews [9,10] emphasize the need for an effective way to work with patient data when modelling computer-interpretable guidelines: They state that there is a lack of a standard way to represent patient data and achieve integration with electronic health records (EHRs). Therefore, effective automatic guideline execution in healthcare needs EHRs that facilitate guideline-oriented CDS, i.e. facilitate the effective modelling and execution of computer-interpretable guidelines.

Furthermore, in the particular case of guideline-oriented CDS that is based on rules, a recent study shows the lack of rule languages that allow for shareable and standardised rules in healthcare [11]. As the computerisation of guidelines often involves their representation as rules, that finding calls for health information systems that are based on standards, both when it comes to their EHRs and CDS components.

EHRs deployed in healthcare vary in their ability to provide CDS, e.g. different countries have reached different levels of satisfaction of this EHR-CDS combination [12]. Nevertheless, most EHRs have not reached a level of sophistication that is satisfactory for executing guidelines effectively, which is important for supporting evidence-based medicine properly. The reasons for this deficiency include

– the separate evolution of EHR and CDS methodology historically,

– the lack of EHRs that fulfill minimal requirements specified by bodies like ISO [13], e.g. the requirement to use standard terminologies and information models,

– the lack of maintainability of and interoperability between CDS components in EHRs and

– the lack of EHR semantics that capture the fine-grained and highly structured patient data needed by computerised guideline execution, e.g. data about any diagnosis of head trauma in the last three years for a particular patient, irrespective of the diagnosing healthcare institution, or data that provide the exact amount of tobacco consumption for a certain patient no matter in which clinical setting the data were recorded.

To solve the latter issue, many efforts are underway to achieve semantically well-defined clinical models for providing necessary data types and facilitating terminology bindings. Concepts supporting healthcare semantics definition include reference information models for defining relevant data types, archetypes for capturing knowledge content and terminology collections [14].

These semantic EHR efforts include openEHR, ISO 13606, Health Level Seven Reference Information Model (HL7 RIM), Integrating the Healthcare Enterprise (IHE) and Clinical Content Models (CCM), which are all working on data exchangeability in their EHR solutions and effective clinical content modelling [14]. Moreover, recent efforts like the Clinical Information Modeling Initiative (CIMI) are attempting to reach a consensus amongst some of the above mentioned different approaches to clinical content modelling [15].

The openEHR specifications, which we use for the research presented here, offer a two-level modelling approach that separates clinical knowledge from information modelling; the former is captured in openEHR archetypes while the latter is done using a standard information model [16]. This means that clinical requirements are developed independently of software and leads to the possibility of understanding the same set of clinical data across EHRs that have different software architectures. Furthermore, openEHR’s Archetype Query Language allows for data queries on the fine-grained level needed by guidelines. Therefore one of the most attractive prospects of combining openEHR with computerised guidelines is achieving exchangeable CDS components, so that once some CDS functionality is developed, it can be used by any other party using openEHR.

The problem still remains that none of the semantic EHR initiatives mentioned above offer thorough guideline computerisation functionality, which would need further consideration of guideline logic, process and workflow aspects. Some solutions have been proposed that combine openEHR and CDS methods: Chen et al. proposed a new method of representing guidelines using openEHR archetypes, openEHR templates and rules [17], Lezcano et al. also pursued combining openEHR and rules, in which they further added the web ontology language (OWL) to the equation [18] and Barretto studied incorporating CDS aspects directly into the openEHR specifications [19].

This study combines openEHR and guideline computerisation based on rules. We already represented guidelines using semantic concepts provided by openEHR [20]. Now we go from modelling to application by running a technology that relies on an openEHR-based guideline representation model – the Guideline Definition Language (GDL), a formalism very recently authored and added to the openEHR specifications [21]. GDL allows combining openEHR archetypes with rules and adding different clinical terminologies as well as human languages. GDL can be used intuitively if one is familiar with ADL, the Archetype Definition Language (cf. [22]).

Our aim is to explore experimentally the practicability of GDL and its underlying archetype structure by using tools that facilitate this technology and with the help of fictitious patient data. Also, we aim to report on the artefacts produced by this new technological approach in this particular experiment. We explore the computerised retrospective checking of compliance with clinical practice guidelines for acute stroke care through executing compliance checking rules written in GDL. Such research can contribute to the exchangeability of guideline-oriented CDS functionality between different health information systems through their EHRs and thereby facilitate the effective implementation of CDS systems. Sharing of computer-interpretable guidelines (CIGs) has also recently been identified as an important, though often forgotten, step in the ‘CIG life-cycle’ [23].

## Methods

### Choice of guidelines

We chose the clinical domain of acute stroke care to drive and test our approach. The knowledge we gathered about acute stroke care guidelines comprises knowledge from the European ‘guidelines for management of ischaemic stroke and transient ischaemic attack’ [24] as well as updates to those based on ground-breaking new evidence [25,26], knowledge of thrombolysis contraindications, where thrombolysis is a decisive treatment option within acute stroke care, and knowledge of calculating the National Institutes of Health Stroke Scale (NIHSS) score. A further motivation for the choice of working with thrombolysis contraindications is that contraindications for a certain treatment seem generally suitable for making retrospective compliance checks, as contraindications are typically formulated in more concise terms than recommendations in guidelines’ text. Thus we mainly focused on thrombolysis contraindications.

### Modelling of guidelines

**Figure 1:**
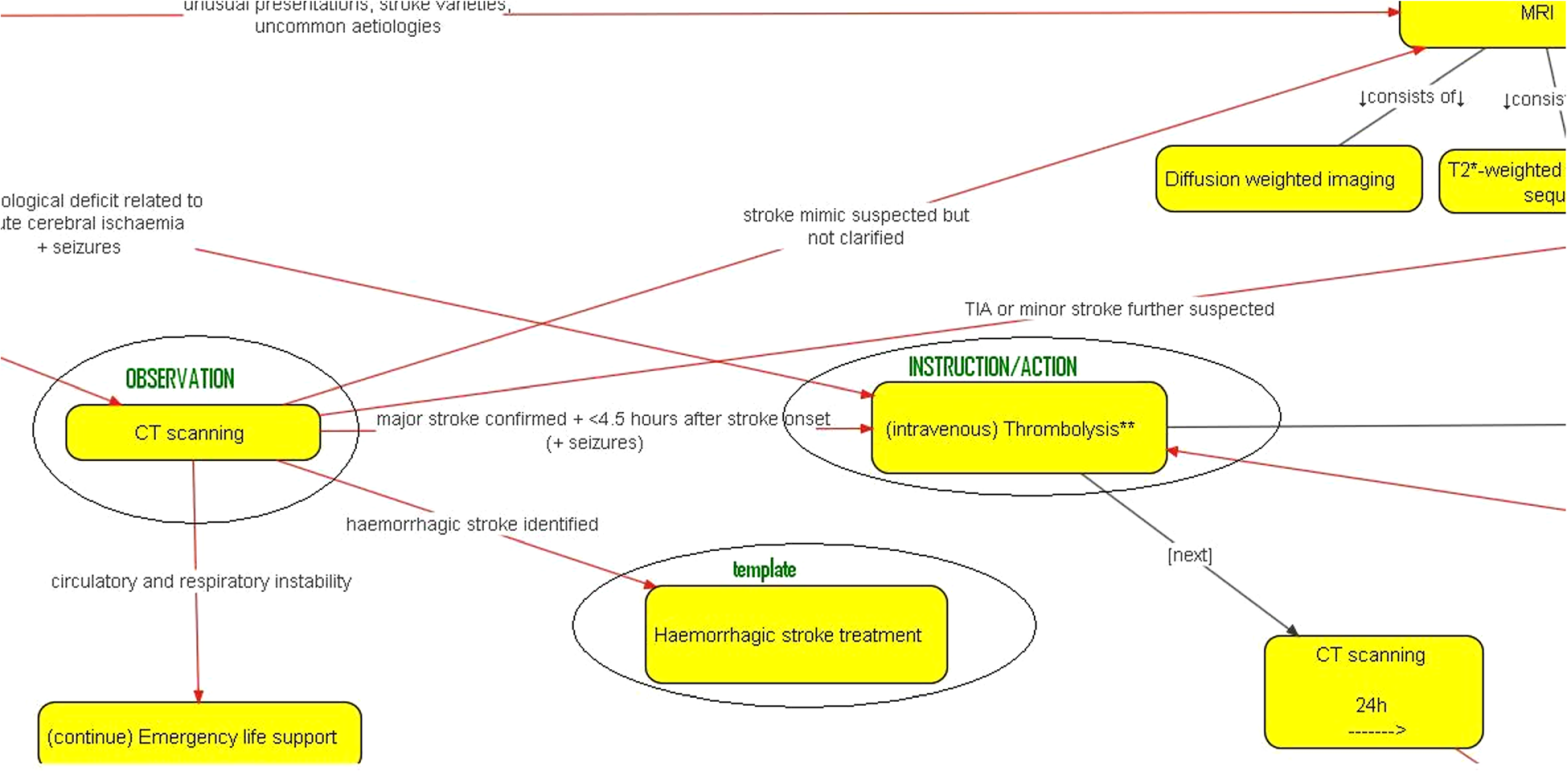
European acute stroke management guidelines generically represented with a focus on openEHR compatibility.

NA interviewed TPM, a stroke physician and researcher, extensively for obtaining an understanding of the European guidelines for acute stroke management. The interviews led to a generic (guideline language-independent) representation of those guidelines on the basis of openEHR semantic concepts, which we recently published [20]. The procedure described in [20] begins with clarifying misunderstandings a medical informatician might have about guideline recommendations, and then aims at creating a chronological order of the activities that happen within the guideline processes. The activities according to this methodology correspond to openEHR CARE_ENTRY classes, i.e. OBSERVATIONs, EVALUATIONs, INSTRUCTIONs or ACTIONs, or openEHR templates. The activities are connected by either guideline conditions (e.g. ‘NIHSS score > 30’), part-whole relationships (e.g. monitoring consists of blood pressure, temperature and oxygen saturation monitoring) or their mere chronology, i.e. activities not designated with the former two relationships happen after each other chronologically. Later activities are further to the right and earlier activities further to the left (e.g. CT scan after thrombolysis in the guidelines leads to CT scan being to the right of thrombolysis). The idea with such a representation is to facilitate a smooth transition to executing guidelines in openEHR-based systems, be guideline representation model-independent and provide a basis for guideline verification between knowledge engineers or medical informaticians and physicians. Figure 1 shows an extract from our generic representation of the European acute stroke management guidelines.

We also used interviews with TPM to clarify knowledge gaps and misunderstandings about thrombolysis contraindications, which was necessary to be able to reach computable expressions of the contraindications. This clarification need typically arises when it comes to representing temporal aspects or diagnoses stated in abstract terms that are usually only clear to clinicians.

A stroke clinical research team at Karolinska Institutet and Karolinska University Hospital in Stockholm, Sweden provided us with the list of thrombolysis contraindications, which are in line with the European stroke guidelines we used and recent updates to those.

The following section shows the thrombolysis contraindications, which we partly refined (see above). These are typical examples of criteria that can be used for retrospective non-compliance checking.

#### Contraindications for using thrombolytic treatment of acute stroke

• Stroke onset more than 4.5 hours ago

• Symptom presentation suggesting another aetiology than that of stroke and/or the patient recovered within 30 minutes

• Unclear stroke symptoms

• National Institutes of Health Stroke Scale (NIHSS) score higher than 25

• CT scan shows haemorrhage

• CT scan shows major stroke that covers more than 30% of the middle cerebral artery

• Blood glucose is lower than 3 mmol/litre or higher than 22 mmol/litre

• Blood pressure is higher than 185/110 mmHg despite two attempts of intravenous beta-blocking bolus treatment (approximately 20 mg of Labetalol per bolus)

• History of cerebral haemorrhage or intracranial bleeding

• Patient describes an explosive headache (that resembles a subarachnoid haemorrhage)

• Ongoing or recent severe haemorrhage (extracranial or intracranial)

• Likely postictal paresis

• Suspected septic shock

• Bleeding disorder or anticoagulation treatment

• One of the following: infectious endocarditis, pericarditis, ventricular thrombosis, atrial septal aneurysm, severe heart failure, pancreatitis, severe liver damage

• One of the following in the last week: lumbar puncture, central venous catheter

• One of the following in the last month: operation/biopsy from parenchymatous organs, trauma with internal injuries, duodenal ulcer, bleeding from the urinary tract

• One of the following in the last three months: stroke, head trauma, operation in the central nervous system, definite gastrointestinal bleeding

• Pregnancy, childbirth in the last month, breastfeeding (relative contraindications)

Beside thrombolysis contraindications, i.e. non-compliance criteria, we also used a number of further compliance criteria that we derived from the European stroke guidelines, e.g. criteria evaluating whether a request for MRI was justified, whether a patient’s body temperature was monitored correctly for pyrexia (fever) or whether a patient’s too low oxygen saturation was acted upon and how. The following section presents some of these criteria that can be used for retrospective compliance checking.

#### Compliance criteria derived from the European guidelines for ischaemic stroke and transient ischaemic attack management

• An MRI of the brain is justified if the patient’s neurological examination reveals posterior circulation stroke varieties or uncommon aetiologies.

• An MRI of the brain is justified if the patient’s CT scan leads to suspecting a stroke mimic but does not rule it out.

• Thrombolysis needs to be performed if the neurological examination of the patient reveals a deficit related to acute cerebral ischaemia within 4.5 hours as of stroke onset.

• Body temperature should be monitored within the first 72 hours after stroke onset for values that exceed 37.5 degrees Celsius.

• If oxygen saturation is below 95% then oxygen should be administered.

### Computerisation of guidelines

The Guideline Definition Language (GDL) is a declarative formalism very recently authored and added to the openEHR specifications [21]. GDL allows combining openEHR archetypes with rules and adding different clinical terminologies as well as human languages. GDL is neutral to any reference terminology as locally defined (coded) terms instead of external terminology codes are used by GDL rules, enabling modification of the term codes without changing the rule definition [21]. GDL can be used intuitively if one is familiar with ADL, the Archetype Definition Language (cf. [22]).

Based on the representation in Figure 1 and our refined thrombolysis contraindication expressions, it was straightforward to extract the corresponding rules. We used GDL in order to create rules that are well connected to a standard EHR approach and standard clinical terminologies. The standard EHR approach consisted of openEHR archetypes and the openEHR reference information model (openEHR RM). Bindings to any terminologies of choice were possible through GDL. We used the Systematised Nomenclature of Medicine Clinical Terms (SNOMED CT) [27], the International Classification of Diseases in its 10^th^ version (ICD-10) [28] and Anatomical Therapeutic Chemical (ATC) codes [29].

The following illustrates the procedure we used to computerise the guidelines with GDL, using the thrombolysis contraindication ‘National Institutes of Health Stroke Scale (NIHSS) score higher than 25’ (cf. section 'Modelling of guidelines' above. What our GDL representation needs in this case is a data type from the openEHR reference information model (openEHR RM) that represents a count – DV_COUNT, and for that to be obtained in the knowledge context of the NIHSS score in the form of an archetype. So we compare the score DV_COUNT element from an appropriate NIHSS OBSERVATION archetype (we authored this archetype in this case because it was not available in the common repositories) with the threshold value of 25. This way we have accounted for the CDS input part. Our CDS output would in this case be setting a DV_BOOLEAN value of a thrombolysis contraindications EVALUATION archetype (also self-authored) to true in case the NIHSS score exceeds 25 and false if it does not. The corresponding GDL lines of code would be

when = <"$gt0008>25",…>

then = <"$gt0016=true",…>,

where gt0008 and gt0016 are codes that simply refer to the data elements mentioned above and had been defined earlier in the GDL code from the respective archetypes.

Similarly, the conditions and actions in Figure 1 can be represented using the appropriate GDL expressions and archetype data elements and in those examples, terminology bindings are also often present due to the existence of several diagnoses or conditions such as ‘stroke’ or ‘stroke mimic’. For example, a stroke diagnosis could be represented by both the SNOMED CT concept ID 230690007 and the ICD-10 code I64. In GDL, the existence of this diagnosis would be checked using codes as above (also codes to represent the particular diagnosis as opposed to the relevant diagnosis archetype data element), which would then be connected to the different terminologies in GDL’s terminology binding section, e.g.

when = <"$gt0003 is_a local::gt0102|stroke|",…>

[other code before the terminology binding section]

["ICD10"] = (TERM_BINDING) <

bindings = <

["gt0102"] = (BINDING) <

codes = <[ICD10::I64],…>

[rest of ICD-10 section]

["SNOMED-CT"] = (TERM_BINDING) <

bindings = <

["gt0102"] = (BINDING) <

codes = <[SNOMED-CT::230690007],…>.

The Results section goes into more detail regarding the produced GDL rules and connects them to the additional files we deliver together with this article.

### Execution of guidelines on patient data

We reviewed one real patient case in order to get a good understanding of what realistic patient data look like, e.g. realistic blood glucose values or patient history notes. Data from the patient case then guided us in developing a Java program that generated random acute stroke care data, which we used to populate our experimental environment. We created 49 patient cases which had different features that could be tested for compliance.

We stored the patient cases using the Data Archetype Definition Language (dADL) [22] and ran the guidelines represented in GDL on these patient cases.

### Manual validation of compliance results

In order to validate the automatic compliance results we got, we went through the 49 mock patient cases manually to check their compliance with the stroke guidelines we used, and compared the correct results of the manual inspection with the compliance results achieved by our computerised CDS logic.

### Tools used

We utilised the open-sourced GDL Editor for authoring GDL files and the CDS Workbench for running them on patient cases, where the CDS Workbench also facilitated creation of patient data based on openEHR archetypes. Where available archetypes from the international archetype repository [30] were not sufficient, we used the open-sourced Ocean Archetype Editor to create new archetypes or modify existing ones.

**Figure 2:**
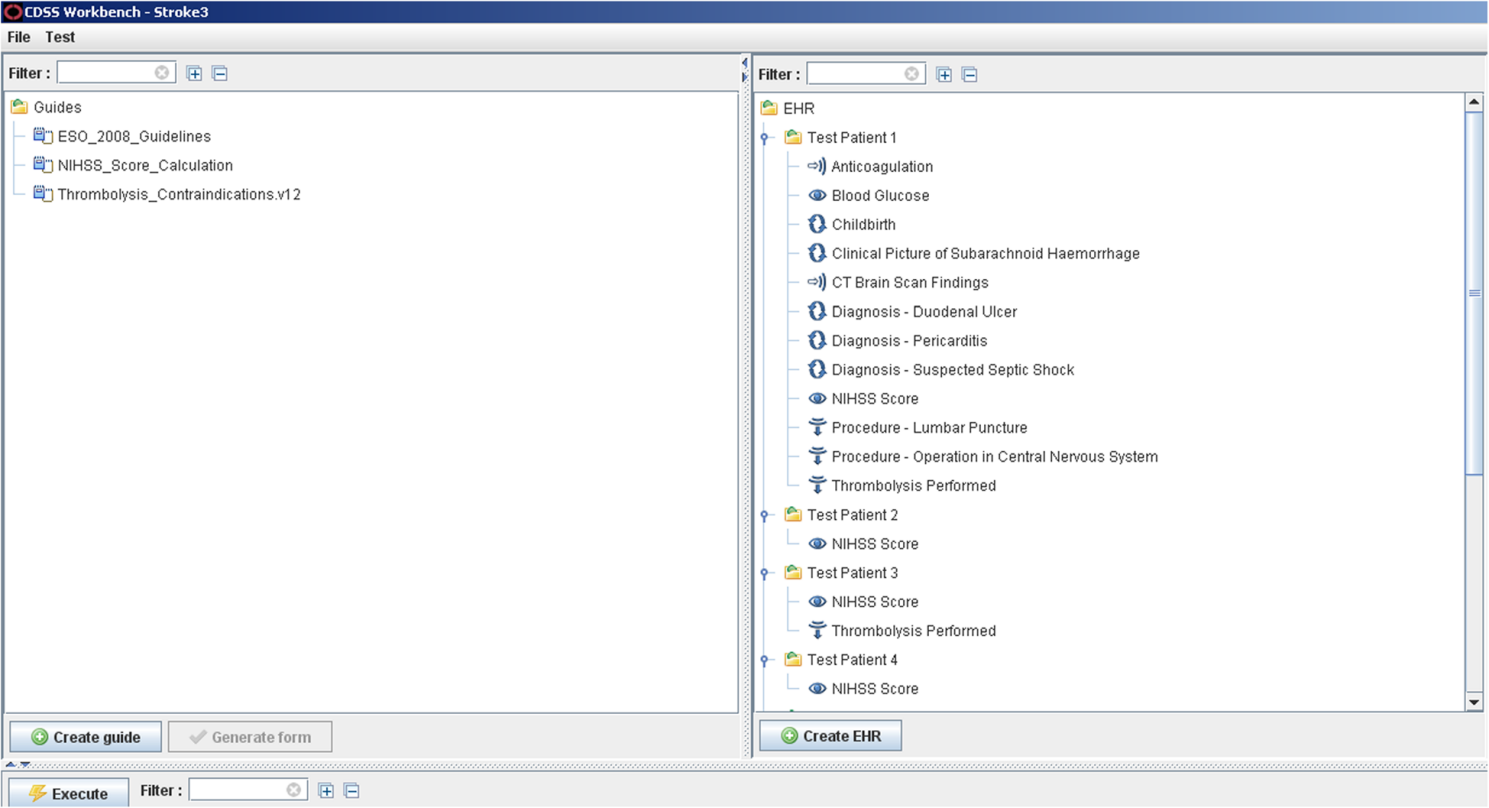
CDS Workbench.

Additional file 1 has the URL of the GDL website, with the possibility to download installation files of the GDL Editor. Figure 2 shows the CDS Workbench.

The GDL Editor provides a certain level of terminology integration by harnessing the built-in terminology service. It is possible, for example, to search for a term by a string partial match and navigate hierarchies in ICD-10 and ATC. But in order to achieve a higher level of integration with more sophisticated terminology resources such as SNOMED CT, a full-fledged terminology service will have to be used. The GDL Editor does not currently support archetypes based on different information models than the openEHR RM.

### Ethical considerations

Data from one patient case, which had been collected as part of usual care, were anonymised following the patient’s informed consent. The patient authorised publication of her/his anonymised data upon review of the present manuscript. We did not, however, use these anonymised data one to one, but based our virtual patient cases on them (cf. section ‘Execution of guidelines on patient data’ above).

### Technical set-up summed up

In accordance with the methods we chose for computerising and applying guidelines, our technical environment consisted of

• GDL rules to represent guideline knowledge,

• openEHR archetypes to handle clinical knowledge elements,

• the openEHR RM to handle archetyped clinical data elements,

• bindings to the terminologies SNOMED CT, ICD-10 and ATC and

• the dADL format for storing patient data.

### Methods and materials summed up

Table 1 shows this study’s methods and materials while Table 2 aligns some of those materials and methods further by connecting the tasks we went through with the methods or tools we used to achieve those tasks.

**Table 1:** Methods and materials

**Methods**	**Materials**
Guidelines	European acute stroke guidelines, thrombolysis contraindications, NIHSS score calculation
Computerised guidelines in GDL using openEHR archetypes and terminology bindings	GDL Editor, international archetype repository, Archetype Editor
Compliance checking of mock patient cases in dADL format	CDS Workbench
Manual validation of compliance results	Support of expert physician

**Table 2:** Alignment of tasks done in this study with tools or methods used to achieve them

**Task**	**Tools/methods used**
Guideline understanding and transformation into generic representation	Verification with expert physician and the openEHR-oriented representation method developed by the authors (cf. Figure 1 and [20])
Collecting archetypes to satisfy the stroke guidelines’ knowledge and data needs	International archetype repository (to retrieve existing archetypes) and Ocean Archetype Editor (to create new archetypes)
Transforming guidelines into a computer-interpretable openEHR-based format	GDL (combines rule logic and archetypes) facilitated by GDL Editor
Binding guideline terms to standard terminologies like SNOMED CT and ICD	GDL facilitated by GDL Editor
Testing GDL rules (executing them on test values)	GDL Editor
Executing GDL rules on patient data in dADL format	CDS Workbench
Producing patient cases in dADL format	CDS Workbench
Producing compliance statistics based on GDL rule execution on patient cases	CDS Workbench
Manual validation of compliance results	Judgement of expert physician

## Results

Through the example of clinical practice guidelines for acute stroke management, we explored the use of GDL in combination with openEHR to perform automatic guideline compliance checking in an experimental setting.

The following details the compliance checks, their manual validation and the different artefacts produced herein.

### Compliance checking

**Figure 3:**
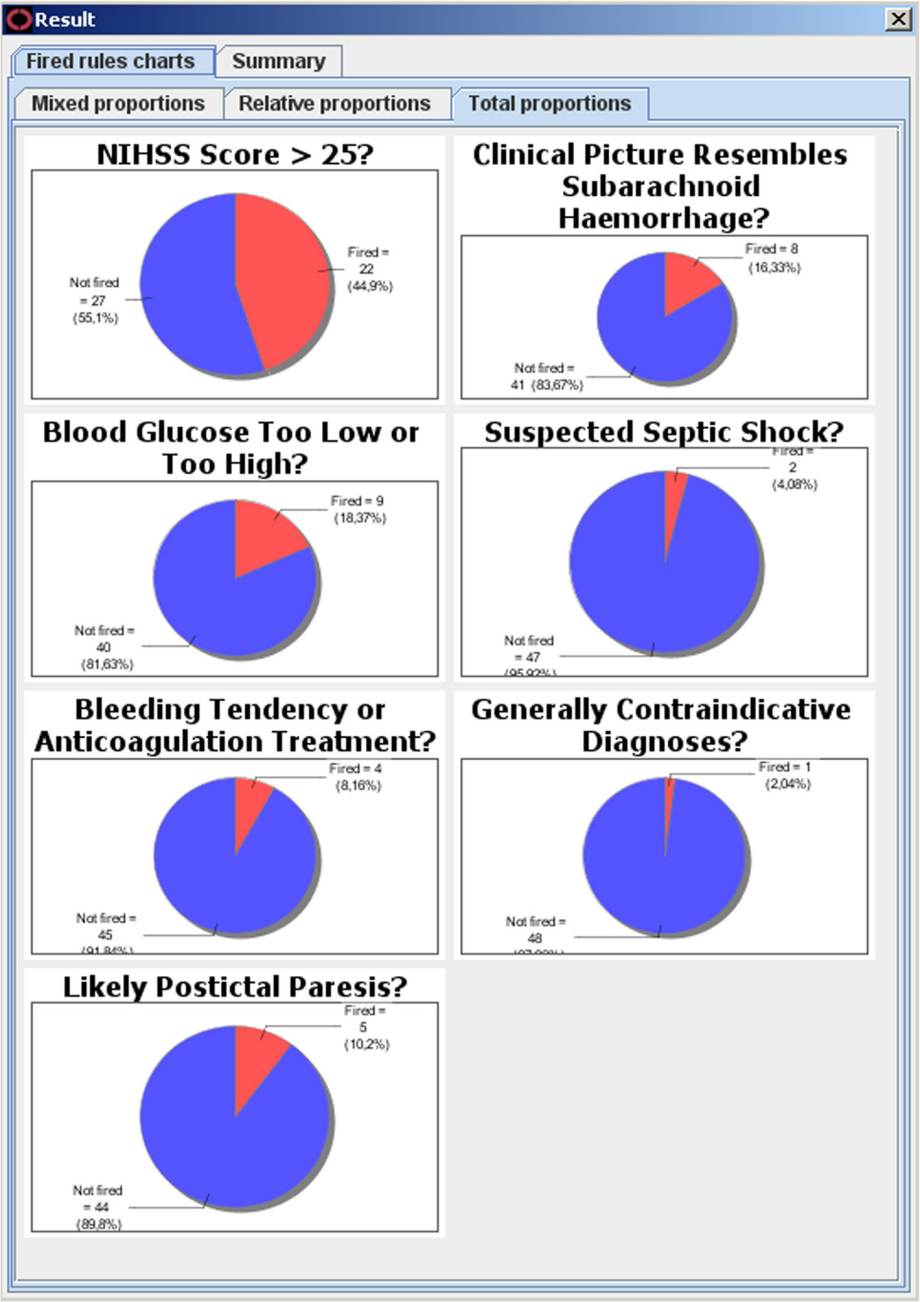
Compliance statistics.

We authored GDL files that contain the guideline knowledge and logic of our chosen acute stroke guidelines. Then we ran the GDL file related to thrombolysis contraindications on patient cases in dADL format and validated the results manually. This manual validation of the 49 mock patients confirmed the results of the automatic compliance checking. Figure 3 shows an example of the statistics we obtained automatically from the CDS Workbench, with different compliance criteria and their fulfillment.

Table 3 is a repetition of the list under the section 'Contraindications for using thrombolytic treatment of acute stroke' in Methods, but also shows which rules we represented in GDL and which we did not. The five rules we did not represent in GDL pose a challenge due to some vague expressions such as ‘unclear’ and ‘major’ as well as missing temporal aspects such as the time interval between the ‘two attempts of intravenous beta-blocking bolus treatment’ or in ‘recent severe haemorrhage’. Although TPM provided explanations regarding the exact meanings of these rules, we decided that representing them in an objective manner would need consensus from several clinical experts. A higher level of detail in those rules should allow their representation in GDL.

**Table 3:** Thrombolysis contraindications achieved by GDL rules

**Thrombolysis contraindication**	**Represented in GDL (yes/no)**
Stroke onset more than 4.5 hours ago	Yes
Symptom presentation suggesting another aetiology than that of stroke and/or the patient recovered within 30 minutes	No
Unclear stroke symptoms	No
National Institutes of Health Stroke Scale (NIHSS) score higher than 25	Yes
CT scan shows haemorrhage	Yes
CT scan shows major stroke that covers more than 30% of the middle cerebral artery	No
Blood glucose is lower than 3 mmol/litre or higher than 22 mmol/litre	Yes
Blood pressure is higher than 185/110 mmHg despite two attempts of intravenous beta-blocking bolus treatment (approximately 20 mg of Labetalol per bolus)	No
History of cerebral haemorrhage or intracranial bleeding	Yes
Patient describes an explosive headache (that resembles a subarachnoid haemorrhage)	Yes
Ongoing or recent severe haemorrhage (extracranial or intracranial)	No
Likely postictal paresis	Yes
Suspected septic shock	Yes
Bleeding disorder or anticoagulation treatment	Yes
One of the following: infectious endocarditis, pericarditis, ventricular thrombosis, atrial septal aneurysm, severe heart failure, pancreatitis, severe liver damage	Yes
One of the following in the last week: lumbar puncture, central venous catheter	Yes
One of the following in the last month: operation/biopsy from parenchymatous organs, trauma with internal injuries, duodenal ulcer, bleeding from the urinary tract	Yes
One of the following in the last three months: stroke, head trauma, operation in the central nervous system, definite gastrointestinal bleeding	Yes
Pregnancy, childbirth in the last month, breastfeeding (relative contraindications)	Yes

### Technological components of GDL applied to acute stroke

#### openEHR archetypes based on the openEHR reference information model

In [20] we identified and authored archetypes that are needed to capture the clinical knowledge in the acute stroke care setting, but did not list those archetypes there as the focus of that study was to highlight the methodology used and its implications. We append the list of those archetypes to this article in Additional file 2. Given the compliance criteria we had put together, we chose the archetypes required to represent the compliance data from the list above.

Our archetypes are based on the openEHR reference information model (openEHR RM) and are of the CARE_ENTRY type, i.e. OBSERVATIONs, EVALUATIONs, INSTRUCTIONs and ACTIONs (cf. [16]).

**Figure 4:**
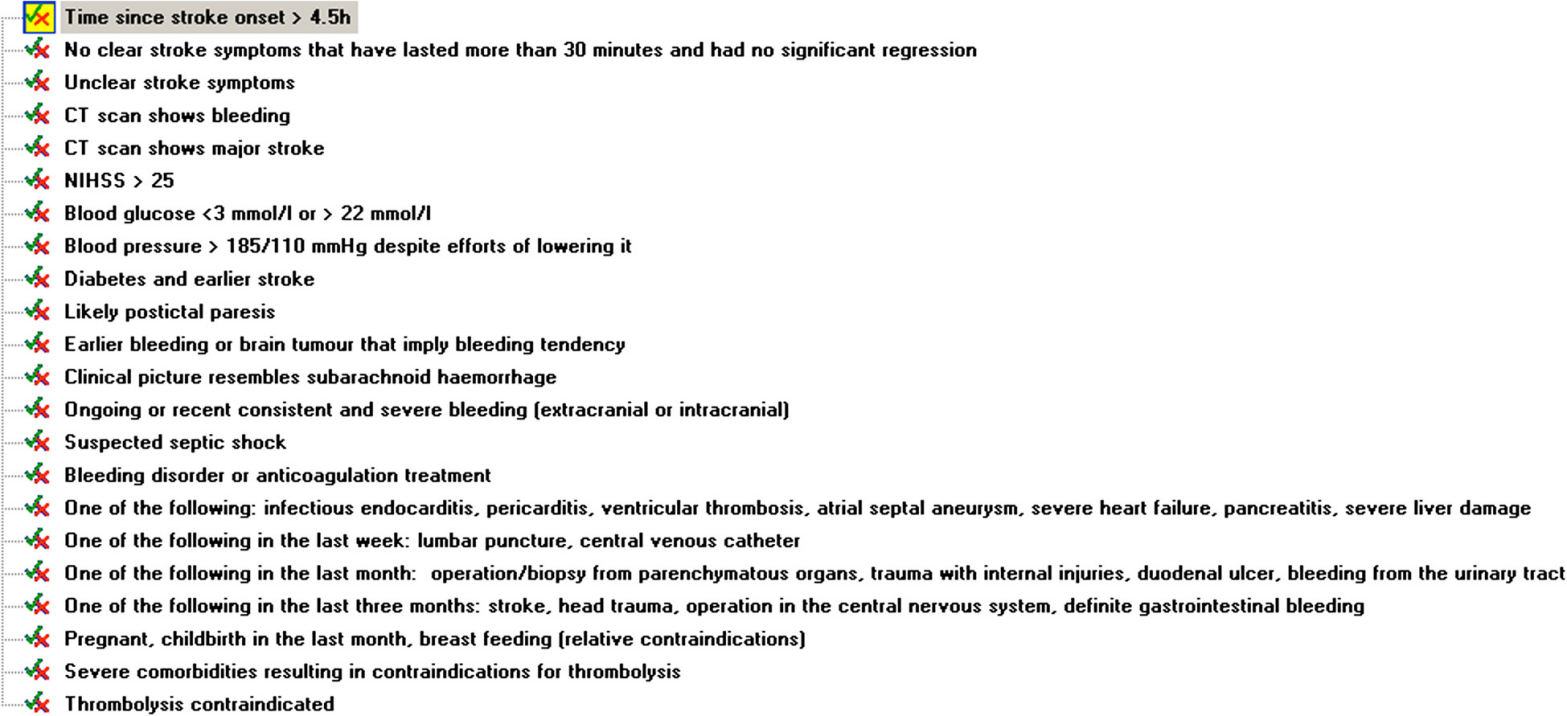
Thrombolysis contraindications EVALUATION archetype.

We reused 14 archetypes. Additionally we authored a new OBSERVATION archetype to represent the National Institutes of Health Stroke Scale (NIHSS) and a new EVALUATION archetype that consists merely of openEHR DV_BOOLEAN (cf. [16]) data elements, in order to capture thrombolysis contraindications. Figure 4 shows a screenshot of this archetype from the Ocean Archetype Editor (a tool for authoring archetypes).

The list of archetypes we used, which are within Additional file 3, is as follows:

– openEHR-EHR-ACTION.intravenous_fluid_administration *(reused)*

– openEHR-EHR-ACTION.procedure *(reused)*

– openEHR-EHR-EVALUATION.alert *(reused)*

– openEHR-EHR-EVALUATION.problem_diagnosis (*extended by a data element called ‘Confidence’, which is a coded text that has the options ‘Suspicion’ and ‘Certainty’, and further modified to allow a more precise timestamp in the data element ‘Date of initial onset’*)

– openEHR-EHR-EVALUATION.thrombolysis_contraindications *(new)*

– openEHR-EHR-INSTRUCTION.imaging *(reused)*

– openEHR-EHR-INSTRUCTION.medication *(reused)*

– openEHR-EHR-ITEM_TREE.gas_administration *(reused)*

– openEHR-EHR-ITEM_TREE.imaging *(reused)*

– openEHR-EHR-ITEM_TREE.medication *(reused)*

– openEHR-EHR-OBSERVATION.blood_pressure *(reused)*

– openEHR-EHR-OBSERVATION.body_temperature *(reused)*

– openEHR-EHR-OBSERVATION.exam *(reused)*

– openEHR-EHR-OBSERVATION.indirect_oximetry *(reused)*

– openEHR-EHR-OBSERVATION.lab_test-blood_glucose *(reused)*

– openEHR-EHR-OBSERVATION.nihss *(new)*

#### openEHR templates based on the openEHR reference information model

openEHR templates enable the use and constraint of data elements from one or more archetypes (cf. [16]). Where needed, we used openEHR templates. This was the case when a certain archetype contained a further archetype (a slot).

#### Guideline definition language rules

We used 16 GDL rules to represent thrombolysis contraindications, 58 GDL rules to represent the National Institutes of Health Stroke Scale (NIHSS) score calculation (setting the different values according to different conditions and calculating the total score) and 6 rules as a sample from the European stroke management guidelines. The three respective GDL files are in Additional file 4.

**Figure 5:**
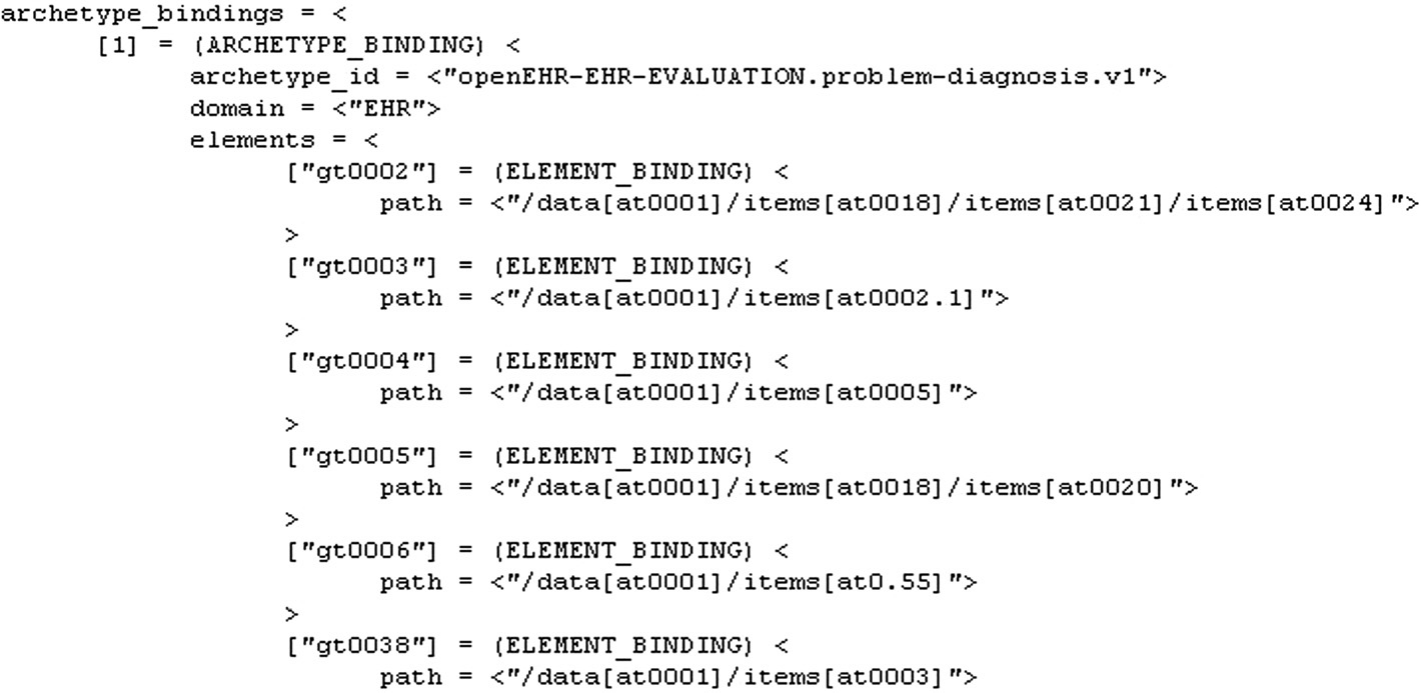
GDL archetype binding for acute stroke care.

**Figure 6:**
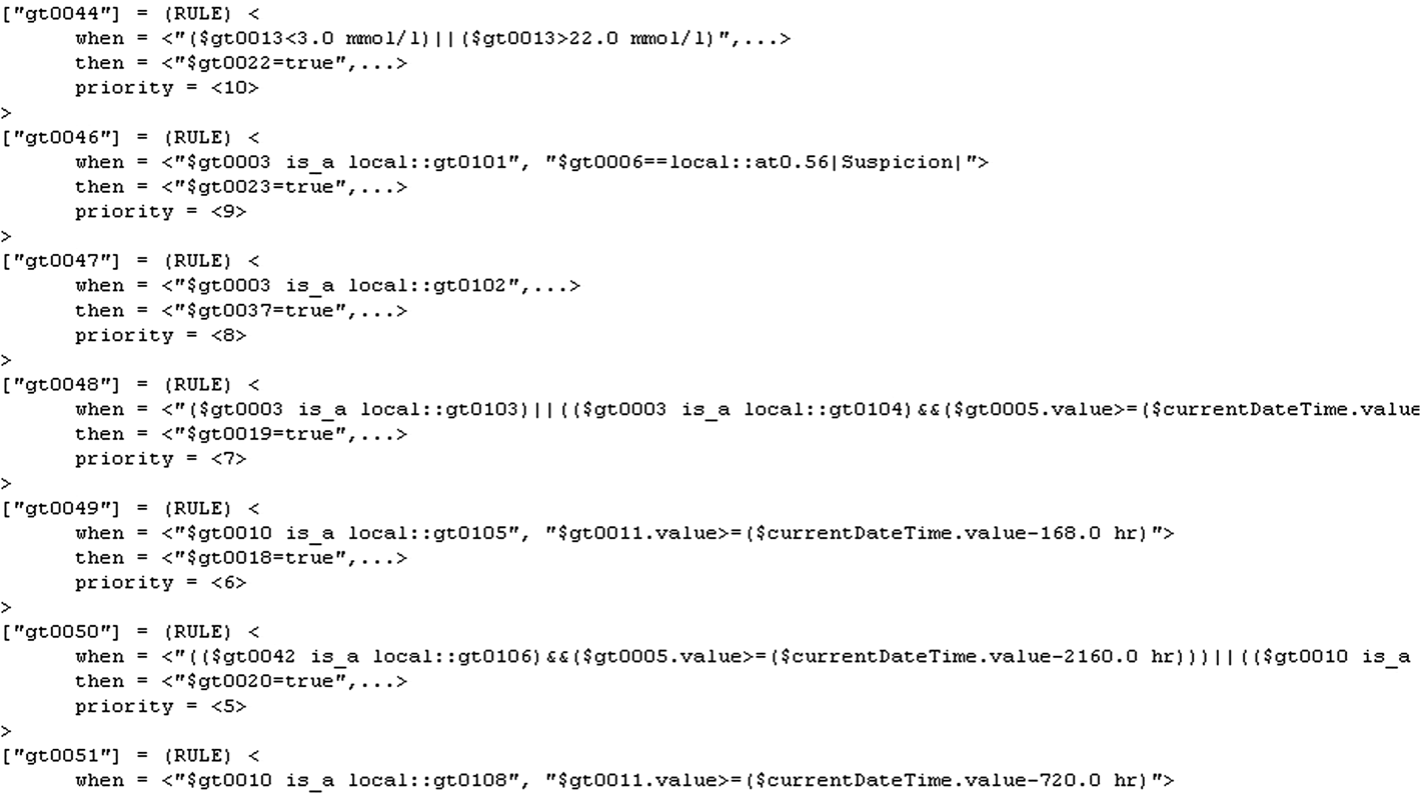
GDL rules for acute stroke care.

**Figure 7:**
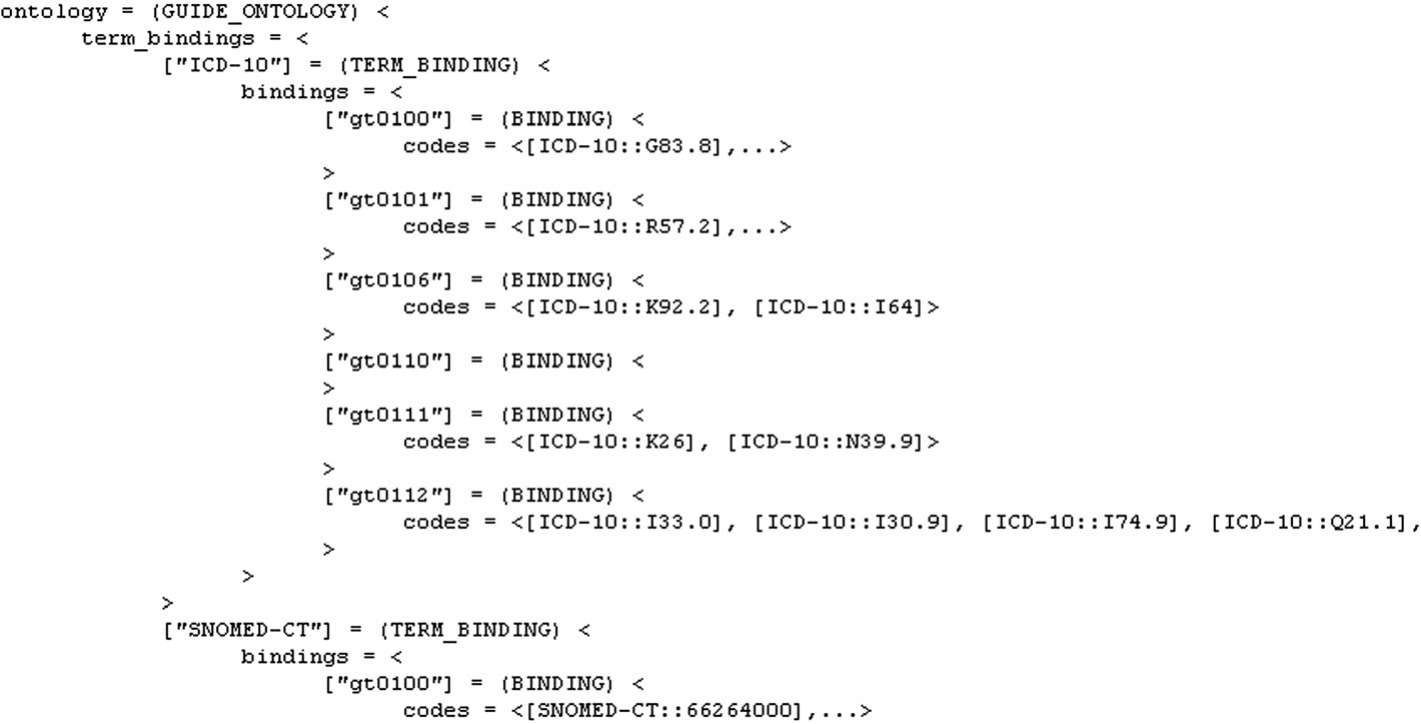
GDL terminology binding for acute stroke care.

As examples of the GDL elements we arrived at in our acute stroke management setting, Figure 5 shows an extract from the archetype binding part of GDL, Figure 6 an extract from the rules section of GDL and Figure 7 GDL terminology bindings.

The archetype binding part in Figure 5 constitutes assigning GDL-specific codes to particular archetype elements needed by the rules of the GDL file of concern, e.g. a GDL file could group together rules related to the same concept, like thrombolysis contraindications. So we import, for instance, the element with the path /data[at0001]/items[at0002.1], which is the diagnosis text, and it is assigned the GDL-specific code gt0003, which is used in the rest of the GDL file to make diagnosis evaluations.

The GDL rules in Figure 6 correspond to our descriptions in the Methods section and so does the terminology binding in Figure 7 (cf. section ‘Computerisation of guidelines’ under Methods).

#### SNOMED CT, ICD and ATC bindings

Figure 7 also shows that we used the Systematised Nomenclature of Medicine Clinical Terms (SNOMED CT) [27] and the International Classification of Diseases (ICD), particularly ICD-10 [28]. Furthermore, we used Anatomical Therapeutic Chemical (ATC) codes [29]. These terminologies allowed us to code diagnoses, procedures and medication-related terms in a standardised manner. Where possible, we coded the same concept with different clinical terminologies simultaneously, which is a feature of GDL. For example, postictal paralysis can be represented both by the SNOMED CT concept ID 66264000 and the ICD-10 code G83.8. Additional file 5 contains a table of the SNOMED CT and ICD-10 codes we utilised within representing thrombolysis contraindications, which contained the large majority of terminology bindings. In total, we used 51 terminology bindings.

#### Patient cases in data archetype definition language format

**Figure 8:**
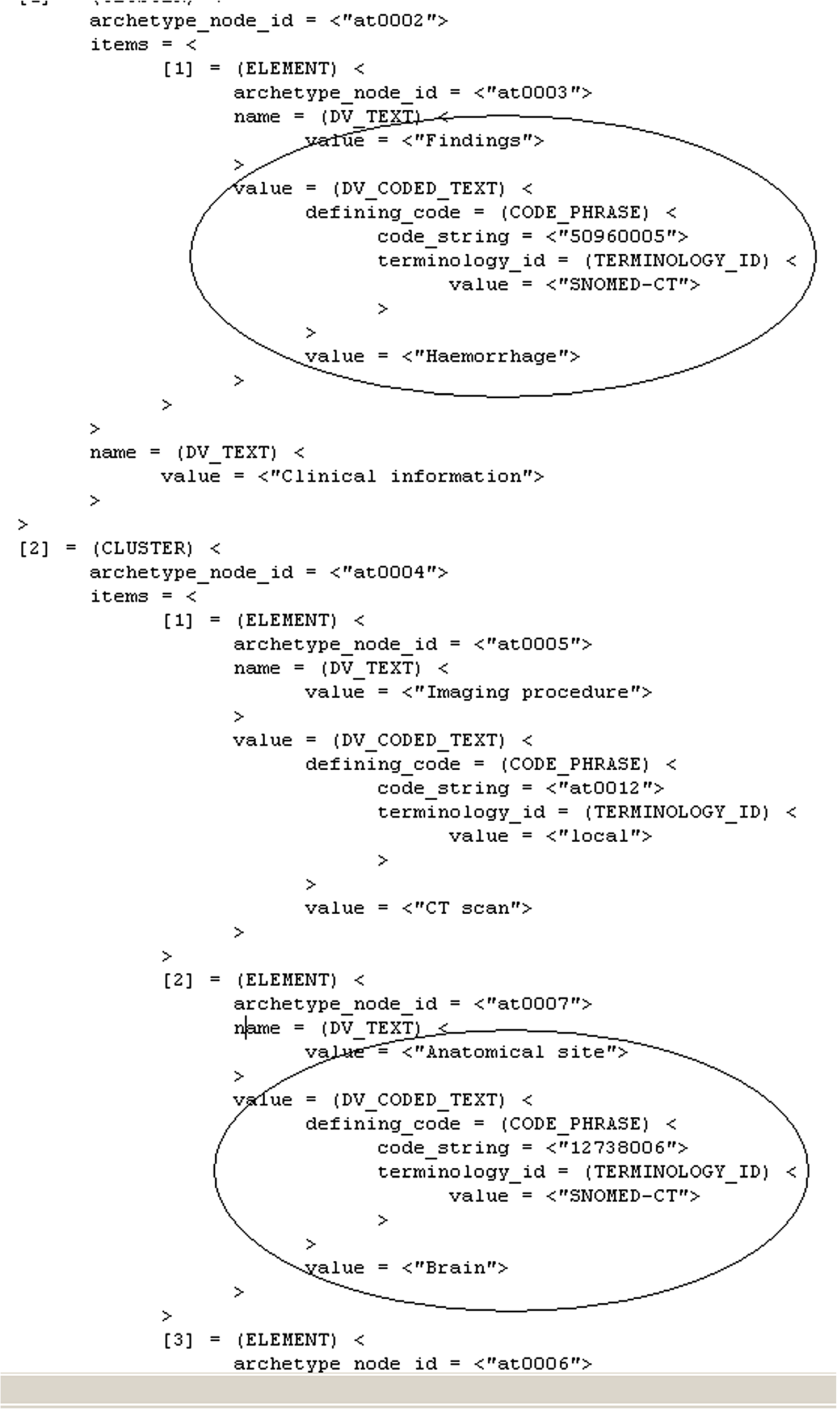
Extract from a patient case in dADL. Data instances of archetype elements are highlighted.

Our 49 patient cases corresponded to 49 dADL files. Figure 8 shows an extract from a dADL file where imaging findings of a patient are recorded. The highlighted parts show how the Findings data element of an imaging archetype is set with the SNOMED CT concept ID 50960005, corresponding to the textual value “Haemorrhage”, and how the Anatomical site data element of the same archetype is set with the SNOMED CT concept ID 12738006, corresponding to the textual value “Brain”.

### Examples illustrating mechanisms of GDL applied to acute stroke

The following examples illustrate GDL mechanisms and rely upon criteria from the section 'Modelling of guidelines' in Methods. These examples and others are available in detail and for hands-on experiences using Additional file 3 together with Additional file 4, or using Additional file 6.

1) Stroke onset more than 4.5 hours ago

Here the value of the data element Date of initial onset of openEHR RM type DV_DATE_TIME from the archetype Problem/Diagnosis is compared using the GDL < operator with a GDL variable called Current Date/Time to check whether stroke onset was within the past 4.5 hours or not. If not, the data element Time since stroke onset > 4.5 hours of openEHR RM type DV_BOOLEAN from the archetype Thrombolysis Contraindications is set to True.

**Figure 9:**
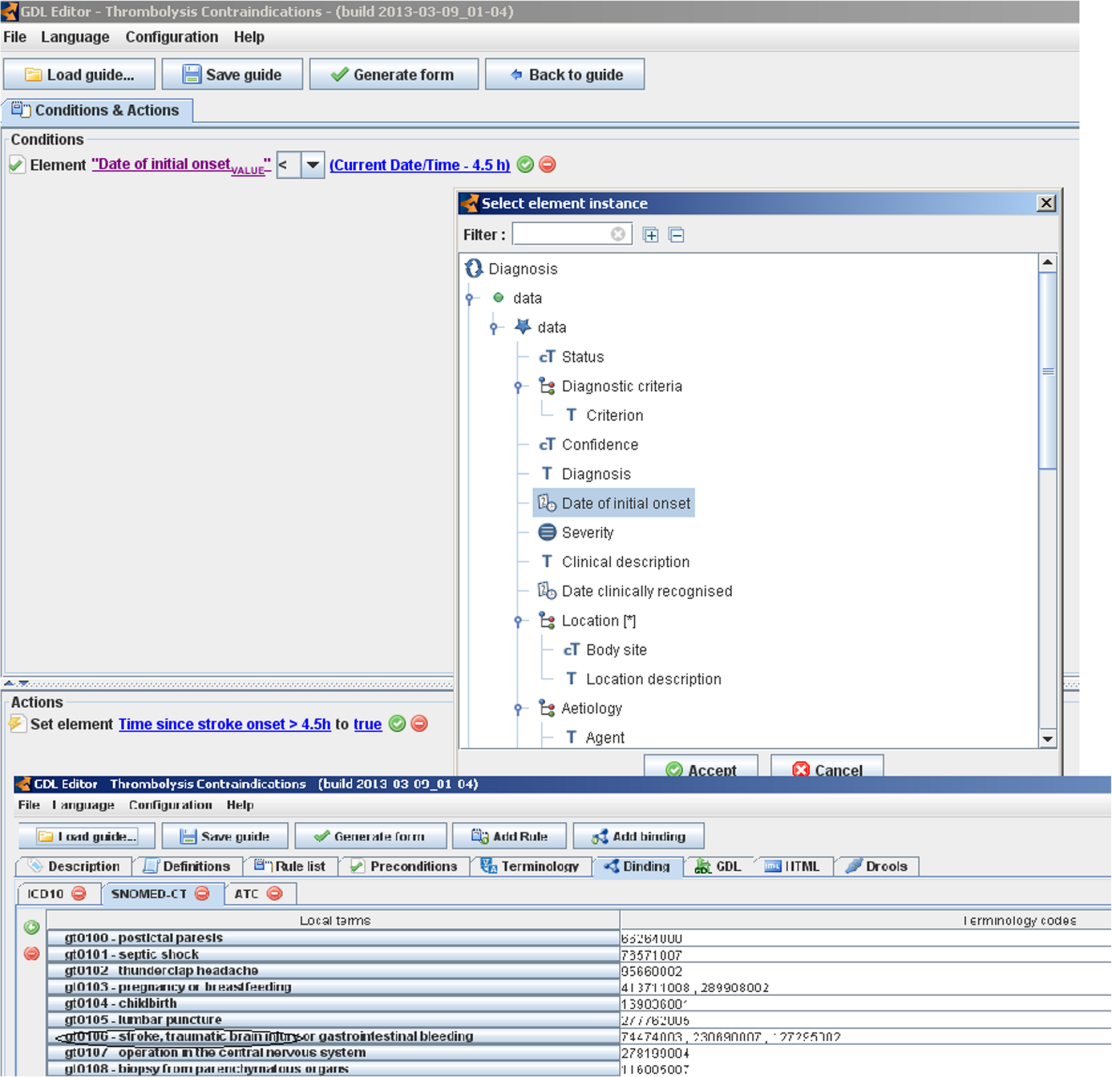
Different aspects of creating a GDL rule in the GDL Editor.

Figure 9 shows the GDL Editor rule view for this rule, in addition to the GDL Editor dialogue that chose the relevant archetype element in the condition and the GDL Editor terminology binding view, where the SNOMED CT part for stroke is highlighted.

2) One of the following: infectious endocarditis, pericarditis, ventricular thrombosis, atrial septal aneurysm, severe heart failure, pancreatitis, severe liver damage

In this GDL rule the value of the data element Diagnosis of openEHR RM type DV_TEXT from the archetype Problem/Diagnosis is checked for belonging to a set of possible diagnoses defined as SNOMED CT and ICD-10 terms simultaneously. If it does fulfill one of the diagnoses, the data element One of the following: infectious endocarditis, pericarditis, ventricular thrombosis, atrial septal aneurysm, severe heart failure, pancreatitis, severe liver damage of openEHR RM type DV_BOOLEAN from the archetype Thrombolysis Contraindications is set to True.

3) One of the following in the last three months: stroke, head trauma, operation in the central nervous system, definite gastrointestinal bleeding

This GDL rule is basically a combination of the GDL functionality in examples 1) and 2) – in that it checks for certain diagnoses or procedures bound to standard terminologies in a specific period of time - complemented by an OR operator to distinguish diagnoses from procedures, the latter represented by the data element Procedure of openEHR RM type DV_TEXT from the archetype Procedure undertaken. Also in analogy to examples 1) and 2), in case the conditions are met a DV_BOOLEAN typed data element from the Thrombolysis Contraindications archetype is set to True.

4) If oxygen saturation is below 95% then oxygen should be administered

The condition here is that the value of the data element SpO2 of openEHR RM type DV_QUANTITY from the archetype Indirect oximetry is checked for being lower than 95% using the < operator. If it is lower, the following actions take place: the data element Gas of openEHR RM type DV_TEXT from the archetype Gas administration is set with the SNOMED CT code for oxygen; the data element Means of delivery of openEHR RM type DV_CODED_TEXT from the archetype Gas administration is set with the archetype-internal code at0006 representing Nasal canula.

## Discussion

We studied the use of openEHR technology in checking compliance with clinical practice guidelines through the example of acute stroke care in an experimental environment. We implemented guideline knowledge and patient cases as instances of the openEHR RM based on openEHR archetypes to retrospectively check compliance with guidelines through openEHR. We thus integrated a semantic EHR technology based on archetypes and a reference information model with automatic guideline execution. Overall, we tested a novel approach to providing exchangeable guideline-oriented CDS components that can be integrated with openEHR-based EHRs.

### GDL in relation to other guideline representation models

Additional file 7 contains a table that uses a couple of selected features to put GDL and other guideline formalisms – languages that create computer-interpretable guidelines - in relation to each other.

This table draws on our own experiences, descriptions by some of the guideline formalisms’ creators [2-5,7,21,31], the review by Isern and Moreno [10], chapter 13 of [8] and a scan of recent literature.

#### GDL vs. GELLO

We consider GELLO [31] to be the counterpart of GDL in the HL7 world. Although comparing GDL and GELLO thoroughly is beyond the scope of this study, the following could be possible points of comparison for future research: expressiveness for CDS purposes, availability of tools and development environments for the two CDS languages, wealth of experiences in their use, quality of documentation and accessibility. The latter criterion made it easier for us to choose GDL in this study, as it is freely accessible like the rest of the openEHR specifications and so is one of its development environments (the GDL Editor has been released as open-sourced software).

Mei et al. report that GELLO was useful within a CDS engine for chronic disease management while criticising the lack of tools that support GELLO use [32]. Koutkias et al. report deficiencies in GELLO’s expressiveness when modelling adverse drug events, could not use GELLO to implement dynamic CDS behaviour where different rules depend upon each other in their execution and notice the lack of a way to represent alerts in GELLO and its CDS information model (the vMR) [33]. A direct comparison with GDL concerning these criteria from this work would not be useful yet, as GDL is in its early stages compared to GELLO and the studies mentioned covered different clinical demands.

### What’s new with this automated compliance checking?

Several research efforts have been published to demonstrate automated retrospective checking of compliance with guidelines, e.g. in [34] and [35]. What is novel in the present work is that it provides a solution based on openEHR, a semantic technology for facilitating semantic interoperability that has been contributing to international standards such as ISO 13606 and receiving attention in industry, academia and nation-wide or regional initiatives of different countries such as Sweden, Brazil and Russia.

### Lessons learned

A reflection regarding availability of clinical knowledge using this technology is that GDL provides a straightforward way of obtaining clinical EHR data elements due to its openEHR-archetypes underpinning. For example, if a rule evaluated whether or not a blood glucose value has exceeded a certain threshold, then all that needed to be done was to access the necessary blood glucose data instance from its corresponding archetype and check its magnitude and unit against the threshold.

Also, GDL allows reuse of archetypes both for reading data values as a part of checking guideline conditions (CDS input) and for setting data values as actions to be taken upon fulfilling guideline conditions (CDS output). For example, if a rule required that an MRI imaging test be ordered (CDS output) if the Glasgow Coma Scale score reaches a certain value (CDS input), then GDL can use the OBSERVATION Glasgow Coma Scale archetype to check its score and the INSTRUCTION imaging archetype to record the imaging order entry.

Furthermore, GDL could be used to enhance openEHR archetypes by uncovering shortcomings they have, leading to a feedback loop from CDS to clinical models (cf. section ‘openEHR archetypes based on the openEHR reference information model’ in Results above).

Deciding whether a component of a GDL rule is going to be an archetype data element or GDL construct is not ambiguous in our experience. GDL constructs are only needed to compare data elements against each other or constant values, check the existence of data elements or set the values of data elements. So the data elements as such were always archetype elements and that was straightforward to derive.

### Stroke limitation

Compared to guidelines from other clinical domains, acute stroke care guidelines are rather straightforward to transfer to computer-interpretable formats. The structure of recommendations in acute stroke care guidelines is often transferrable to simple rules that satisfy basic if-then statements. Guidelines from other clinical domains may have more complex demands on guideline modelling and could thus be more challenging to computerise. We are aware of this limitation, but think that it does not significantly compromise exploring the overall feasibility of combining a semantic EHR technology with rules for automatic guideline compliance checking. Still, there is a need to represent and execute guidelines from other clinical areas in order to further validate GDL’s usefulness for guideline-oriented CDS generally (cf. section ‘Future directions’ below).

### Utilisation of reference information models and standard terminologies

The reviews of different guideline representation models in [9] and [10] emphasize the need for an effective way to work with patient data when modelling computer-interpretable guidelines. They state that there is a lack of a standard way to represent patient data and achieve integration with EHRs. The advantage of our proposal is that it uses the openEHR RM, one of the available reference information models that are meant to standardise data types and data structures in EHRs (there are others like ISO 13606’s reference information model and the HL7 RIM).

The recent study by Zhou et al. [11] shows the lack of ‘rule authoring environments’ that allow for shareable and standardised rules. The rule language for guidelines that we use, GDL, directly tackles that issue by creating rules that are shareable and standardised, as they rely on shareable clinical knowledge content models that tend to get standardised over time in the form of archetypes and use a reference information model for healthcare data in the form of the openEHR RM.

Using other information models such as ISO 13606’s or the HL7 RIM should work with GDL, as long as certain features are provided by the respective information model: data types have to allow set and get operations on them; it should be possible to compare data elements of the same data type; a data element has to be accessible through unique string paths. Having other information models than the openEHR RM as a basis for GDL has not been tested anywhere and there are currently no tools to support that. Archetype data paths, for instance, containing attribute names from openEHR RM classes would hinder executing guideline content directly from archetypes created using other information models. In such cases, mappings between the openEHR RM and other information models would be required.

Additionally, GDL and openEHR archetypes support the utilisation of terminologies such as SNOMED CT and ICD, which further increases shareability and standardisation. Terminology bindings can be vital in providing the different levels of data abstraction or granularity that guidelines often demand. Extensive hierarchies, such as SNOMED CT’s, can be of good help there. For example, it might be important for a guideline to know whether a certain diagnosed disease is a cardiovascular disease. Intra-terminological relations can make it easy to say that ‘Pathological condition X is a cardiovascular disease’, for instance. As mentioned above under the section ‘Tools used’ in Methods, current tools like the GDL Editor need to be developed further to take full advantage of such features.

### Separation of data from rules leads to shareability

We achieved improved shareability of CDS rules through using archetypes, a reference information model and reference terminologies. GDL tackles the well-known curly braces problem mentioned in the background as it separates data – which come from archetypes – from rule logic – provided by GDL expressions.

Furthermore, GDL achieves a separation between rule logic and natural languages (e.g. English, Spanish, Chinese, Arabic, Portuguese…) as well as between rule logic and reference terminologies such as ICD.

### This solution vs. manual compliance checking methodology

Typically manual checking of compliance with guidelines involves checking whether certain conditions evaluate to yes or no, true or false, fulfilled or not fulfilled, i.e. it involves dichotomous or Boolean logic. Checklists often provide the means of Boolean data collection and those data can then be analysed in various ways, as Luker and Grimmer-Somers also show [36]. This sort of compliance checking matches the compliance checking our method performs, where our computerised approach provides all the advantages of automatisation.

However, manual compliance checks can also be more sophisticated. They can involve the derivation of quality indicators and the aggregation of expert opinions as well as various clinical data to obtain compliance scores [37,38]. We did not incorporate such checks into this study, but think that integration of various such metrics would be possible with the same set-up, yielding valuable insights for decision makers in healthcare that may not be possible with merely manual efforts. The inclusion of quality indicators, results of expert opinions and compliance scores will usually rely upon the integration of relatively simple mathematical formulae.

### Rule maintainability and knowledge scalability

There is too little experience with GDL to be able to judge how easily knowledge captured in GDL rules can be maintained and extended. That is a matter that needs evaluation over time. The good news is that there are already some governance models for openEHR archetypes [30], which can possibly be applied to GDL knowledge artefacts too. Furthermore, if these governance models prove successful with archetypes, a part of the rule knowledge would already be maintained, since GDL relies on reusable openEHR archetypes.

Whichever the case, it will always take a considerable amount of time to reach a computable format of the guidelines from their published form, as guidelines tend to be published mostly as narrative text nowadays. Reaching a representation like the one shown in Figure 1, for example, will probably constitute the majority of the time and cost burden involved in maintaining GDL rules. This is due to the time-intensive process of communication between the medical informatician and physician needed for removing vagueness as well as concretizing temporal aspects.

### Closely related studies

There are a couple of research groups that have been working within tightly related research areas to the one this work belongs to. Marcos and Martínez-Salvador [39] developed their own methodology for arriving at the archetypes needed for a certain guideline as well, where their methodology had less of a graphical character than ours in [20] but followed an iterative algorithm for searching archetype repositories and creating new archetypes based on guideline content. Marcos, Maldonado et al. [40] developed a mapping technique to reach interoperability between EHRs and CDS systems that used concepts from the guideline representation model PROforma as well as openEHR together. Their solution had a focus on database data mapping, which had also been the idea behind the knowledge-data ontological mapper (KDOM) developed by Peleg et al. for relational databases in particular [41].

### Future directions

To test the usefulness of GDL for the expression of clinical guideline knowledge in a shareable manner, GDL has to be used to computerise various guidelines from several clinical areas. This would challenge GDL’s ability to represent guideline knowledge in general, which is the primary prerequisite for its success.

Also, GDL seems just as suitable to implement CDS rules to be used at the point of care as it is to implement CDS rules for retrospective compliance checking; the representation of rules in GDL does not differ for those two use cases. However, evaluation studies within clinical practice would be needed to verify at-the-point-of-care CDS through GDL.

When it comes to tools that facilitate working with GDL, there are two obvious demands that tool developers may find interesting to address in the future for purposes of semantic interoperability. The first is the provision of functionality for binding data elements to terms from standard terminologies, e.g. 1) implementing algorithms for pre-coordination and post-coordination using SNOMED CT’s extensive hierarchy, the former helping to find useful relationships (e.g. is-a relationships) between terms in order to avoid the need to manually identify all fitting term concepts, and the latter making it possible to aggregate terms for forming unavailable ones; 2) using lexical and context-based techniques to find suitable terms in a terminology to bind to archetype data elements, as Meizoso García et al. demonstrated with promising success [42]; 3) identifying interesting terms through visualisation techniques [43]. Secondly, current GDL editing and execution tooling does not support using other standard information models than the openEHR RM. Thus, efforts to advance existing GDL tools or create new ones may want to facilitate designing and running GDL content using, for example, the HL7 RIM or ISO 13606’s information model.

Our study fits the context set by Mandl and Kohane in 2012: ’The IT foundation required for health care is the core set of health data types, the formalization of health care workflows, and encoded knowledge (e.g. practice guidelines, decision-support tools and care plans)’ [44].

## Conclusions

We explored computerised retrospective compliance checking with clinical practice guidelines for acute stroke care using semantic EHR technology and thereby contribute to the sharing of computer-interpretable guidelines (CIGs). Particularly, we used a semantic EHR technology that utilises archetypes as well as a reference information model (openEHR RM), and combined it with rules and reference terminologies via the Guideline Definition Language (GDL).

Using the approach presented here, it was possible to automatically check compliance of mock patient cases from stroke care with different guidelines for acute stroke management. This leaves reason to suspect that deploying such medical informatics technology in healthcare practice may ultimately benefit patients and societies. Guidelines from other clinical areas should challenge the proposed solution further.

Our experience with GDL is that it facilitates reuse of archetyped knowledge, utilises archetypes for both rule checking and CDS actions as well as contributes to archetype quality assurance cycles. We acknowledge, however, that further studies – which need to report on expressiveness of GDL, applying GDL to other clinical domains, maturity of GDL-related tools and real-life clinical deployments – are essential for obtaining a comprehensive picture about this new technology’s pros and cons to healthcare stakeholders.

## Abbreviations

ATC: Anatomical therapeutic chemical; CDS: Clinical decision support; dADL: Data archetype definition language; EHR: Electronic health record; GDL: Guideline definition language; guidelines: Clinical practice guidelines; ICD: International classification of diseases; NIHSS: National institutes of health stroke scale; openEHR RM: openEHR reference information model; SNOMED CT: Systematised nomenclature of medicine clinical terms.

## Competing interests

The authors declare that they have no competing interests. Although RC works for the company that developed some of the tools we used in this study (GDL Editor and CDS Workbench), these tools only served the purpose of facilitating parts of our implementation, and did not constitute elements of our studied approach as such.

## Authors’ contributions

NA led the study, collected data, analysed data and drafted the manuscript. RC had the idea for the study, provided expertise in the Guideline Definition Language needed for data collection and analysis, and reviewed different versions of the manuscript. TPM refined all clinical models used in the study, provided clinical as well as medical expertise wherever needed and reviewed different versions of the manuscript. SK conceptualised the methodology of the study and reviewed different versions of the manuscript. All authors read and approved the final manuscript.

## Authors’ information

NA is a doctoral candidate in health informatics at the Health Informatics Centre at Karolinska Institutet in Stockholm. His research focuses on advancing electronic health records and clinical decision support for processes in healthcare.

RC is Chief Medical Informatics Officer at Cambio Healthcare Systems in Stockholm and an affiliated researcher at the Health Informatics Centre at Karolinska Institutet. He holds a PhD degree in medical informatics from Linköping University in Sweden and is well known in the openEHR community for having developed the Java reference implementation of the openEHR specifications. He is a member of the openEHR Management Board and a co-author of the GDL specifications.

TPM works clinically as a neurologist at Karolinska University Hospital and as an associated researcher within the Stroke Research Group of the Department of Clinical Neuroscience at Karolinska Institutet. He holds a PhD degree in experimental neuroscience from Karolinska Institutet.

SK is a professor in health informatics at Karolinska Institutet and Director of the Health Informatics Centre. She holds a PhD degree in medical informatics from the University of Heidelberg in Germany and has contributed to medical informatics research, specifically in the areas of dental informatics, home-based healthcare for the elderly and the intersection between clinical and personal health informatics.

## Pre-publication history

The pre-publication history for this paper can be accessed here:

http://www.biomedcentral.com/1472-6947/14/39/prepub

## Supplementary Material

Additional file 1 GDL URL - includes the URL to the GDL website, where the GDL Editor and related documentation as well as sample files can be downloaded. Click here for file

Additional file 2 Acute Stroke Care Archetype Needs – lists archetypes needed within acute stroke care. Click here for file

Additional file 3 **Archetypes – contains all archetypes used in this work as ADL files.** ADL files can be viewed by the Archetype Editor, available at http://www.openehr.org/downloads/archetypeeditor/home. Click here for file

Additional file 4 **GDL Files – contains all decision support rules created by this work as GDL files.** GDL files can be viewed by the GDL Editor, available at http://sourceforge.net/projects/gdl-editor/. Click here for file

Additional file 5 Thrombolysis Contraindications’ Terminology Codes – shows the SNOMED CT and ICD-10 codes needed within representing thrombolysis contraindications. Click here for file

Additional file 6 GDL Configuration – provides a GDL Editor configuration for running the GDL files from this work; can be used as an alternative to installing and configuring the GDL Editor and run using the ‘startup.bat’ file. Click here for file

Additional file 7 GDL in relation to other guideline formalisms – table that compares GDL to other guideline representation models. Click here for file
