# Supplementary material for: Retrospective checking of compliance with practice guidelines for acute stroke care: a novel experiment using openEHR’s Guideline Definition Language
Source: BMC Med Inform Decis Mak. 2014 May 10;14:39. doi: 10.1186/1472-6947-14-39 (PMC4052843; doi:10.1186/1472-6947-14-39)
Supplement: Additional file 2 — Acute Stroke Care Archetype Needs – lists archetypes needed within acute stroke care. [file 1472-6947-14-39-S2.pdf]

Acute Stroke Care Archetype Needs

Existing	To be Authored
OBSERVATION.blood_pressure* (published)	OBSERVATION.nihss (first draft)
OBSERVATION.body_mass_index* (published)	OBSERVATION.lab_test-C-reactive_protein
OBSERVATION.body_temperature* (published)	OBSERVATION.lab_test-partial_thrombin_time
OBSERVATION.body_weight* (published)	EVALUATION.swallowing
OBSERVATION.height* (published)	EVALUATION.risk-fall_risk_assessment
OBSERVATION.respiration* (published)	ACTION.catheter_insertion (first draft)
EVALUATION.problem* (team review)	ACTION.medication-intra-arterial_thrombolysis
OBSERVATION.ecg* (team review)	OBSERVATION.intracranial_pressure
OBSERVATION.glasgow_coma* (team review)	OBSERVATION.berg_balance_scale
OBSERVATION.heart_rate* (team review)	OBSERVATION.tinetti_scale
CLUSTER.imaging* (draft)	
CLUSTER.anatomical_location* (draft)	
CLUSTER.anatomical_location-precise* (draft)	
ACTION.medication** (team review)	
CLUSTER.amount** (team review)	
CLUSTER.medication_admin** (draft)	
CLUSTER.chemical_description** (draft)	
COMPOSITION.prescription** (team review)	
INSTRUCTION.medication** (team review)	
CLUSTER.timing** (team review)	
ACTION.intravenous_fluid_administration* (draft)	
INSTRUCTION.intravenous_fluid_order* (draft)	
ITEM_TREE.intravenous_fluids* (draft)	
EVALUATION.pregnancy* (draft)	
EVALUATION.excluded-intervention* (draft)	
EVALUATION.exclusion* (draft)	
EVALUATION.exclusion-medication* (draft)	
EVALUATION.exclusion-problem_diagnosis* (draft)	
INSTRUCTION.request* (draft)	
INSTRUCTION.request-lab_test* (draft)	
INSTRUCTION.request-procedure* (draft)	
ACTION.procedure* (draft)	
INSTRUCTION.imaging* (draft)	
ACTION.imaging* (draft)	
ITEM_TREE.imaging* (draft)	
ADMIN_ENTRY.admission* (draft)	
EVALUATION.adverse_reaction* (team review)	

EVALUATION.problem-diagnosis* (team review)	
EVALUATION.reason_for_encounter* (draft)	
EVALUATION.substance_use_summary* (draft)	
CLUSTER.cessation_attempts* (draft)	
EVALUATION.substance_use_summary-alcohol* (draft)	
EVALUATION.substance_use_summary-tobacco* (draft)	
OBSERVATION.exam* (draft)	
OBSERVATION.lab_test* (draft)	
CLUSTER.specimen* (draft)	
CLUSTER.physical_properties* (draft)	
CLUSTER.specimen_preparation* (draft)	
CLUSTER.device* (draft)	
CLUSTER.device_details* (draft)	
CLUSTER.dimensions* (draft)	
CLUSTER.lab_result_annotation* (draft)	
OBSERVATION.lab_test-blood_glucose* (draft)	
OBSERVATION.lab_test-full_blood_count* (draft)	
OBSERVATION.lab_test-urea_and_electrolytes* (draft)	
OBSERVATION.lab_test-esr* (draft)	
OBSERVATION.story* (draft)	
CLUSTER.health_event* (draft)	
CLUSTER.symptom* (draft)	
CLUSTER.symptom-pain* (draft)	
CLUSTER.issue* (draft)	
EVALUATION.clinical_synopsis* (published)	
OBSERVATION.imaging* (draft)	
OBSERVATION.heart_rate-pulse* (draft)	
EVALUATION.alert* (draft)	
EVALUATION.risk* (draft)	
EVALUATION.risk-family_history* (draft)	
INSTRUCTION.non_drug_therapy* (draft)	
ITEM_TREE.gas_administration* (draft)	
OBSERVATION.indirect_oximetry* (published)	
CLUSTER.level_of_exertion* (draft)	
CLUSTER.ambient_oxygen* (draft)	
EVALUATION.goal* (draft)	

* Source: http://www.openehr.org/ckm
** Source: http://dcm.nehta.org.au/ckm

Last update: 2014-01-23
