# Supplementary material for: Retrospective checking of compliance with practice guidelines for acute stroke care: a novel experiment using openEHR’s Guideline Definition Language
Source: BMC Med Inform Decis Mak. 2014 May 10;14:39. doi: 10.1186/1472-6947-14-39 (PMC4052843; doi:10.1186/1472-6947-14-39)
Supplement: Additional file 5 — Thrombolysis Contraindications’ Terminology Codes – shows the SNOMED CT and ICD-10 codes needed within representing thrombolysis contraindications. [file 1472-6947-14-39-S5.pdf]

Thrombolysis Contraindications - SNOMED CT Concept IDs, ICD-10 Codes

(SNOMED CT version 2012-07-31, ICD-10 version 2010)

Term	SNOMED CT Concept ID	ICD-10 Code
Acute heart failure	56675007	I50.0
Administration of medication not done due to contraindication	373147003	
Assessment using National Institutes of Health stroke scale	450742003	
Atrial septal aneurysm	95440004	Q21.1
Biopsy	86273004	
Birth of child	169836001	
Bleeding tendency	64779008	D68.9
Blood glucose level	365812005	
Blood pressure	75367002	
Brain	12738006	
Brain tumour	254935002	
Breastfeeding	413711008	
Central nervous system	278199004	
Central venous catheter	52124006	
Cerebral haemorrhage	274100004	
Computed tomography of brain perfusion	433111008	
Computerised axial tomography of brain with radiopaque contrast	396207002	
Computerised tomography	77477000	
Diabetes	73211009	E14
Duodenal ulcer	51868009	K26
Gastrointestinal bleeding	74474003	K92.2
Haemorrhage	50960005	
Infective endocarditis	233850007	I33.0
Internal injury	79322002	T14.8
Intracranial haemorrhage	1386000	I62.9
Ischaemic stroke	422504002	
Liver failure	59927004	K72.9
Lumbar puncture	277762005	
National Institutes of Health stroke scale score	450743008	
National Institutes of Health stroke scale	450741005	
Operation	387713003	
Pancreatitis	75694006	K85
Parenchymatous viscus	116005007	
Pericarditis	3238004	I30.9
Postictal paralysis	66264000	G83.8
Pregnancy	289908002	
Right ventricular thrombosis	309518001	I51.3

Septic shock	76571007	R57.2
Stroke	230690007	I64
Subarachnoid haemorrhage	21454007	I60
Thrombolysis contraindicated	390910005	
Thrombolysis	51308000	
Thrombosis	439127006	I74.9
Thunderclap headache	95660002	
Trauma	19130008	
Traumatic brain injury	127295002	
Urinary tract finding	249273002	N39.9
Use of anticoagulation	260678004	
