# Supplementary material for: Retrospective checking of compliance with practice guidelines for acute stroke care: a novel experiment using openEHR’s Guideline Definition Language
Source: BMC Med Inform Decis Mak. 2014 May 10;14:39. doi: 10.1186/1472-6947-14-39 (PMC4052843; doi:10.1186/1472-6947-14-39)
Supplement: Additional file 7 — GDL in relation to other guideline formalisms – table that compares GDL to other guideline representation models. [file 1472-6947-14-39-S7.pdf]

**Guideline formalism / Feature**	*Arden Syntax*	*PROforma*	*GLIF3*	*Asbru*	*SAGE*	*GDL*	*GELLO*
**Type**	rule-based language	task-network model	task-network model	task-network model	task-network model	rule-based language	object-oriented constraint language
**Core elements**	medical logic modules	plans, actions, decisions, enquiries	actions, decisions, synchronisations, branches, clinical stages	logic statements	actions, decisions, contexts, routes	archetype bindings, rule expressions, term bindings	OCL-based expressions
**Information and knowledge complexity**	modular organisation of rules through medical logic modules; uses types from HL7; events trigger medical logic modules	arguments can be attached to decision candidates; thorough support for preconditions, postconditions, triggers and scheduling constraints; based on logic programming	focus on guideline nesting; the HL7 RIM provides a thorough clinical information model; can be used in conjunction with an expression language such as GELLO	unique feature of specifying and considering intentions; support for complex temporal patterns in relation to intentions; parameter definitions can be made based on context	various recommendation types available and order sets supported; the HL7 RIM provides a thorough clinical information model; GELLO provided as expression language	archetypes represent clinical knowledge as shareable clinical content models; the openEHR RM provides a thorough clinical information model; term bindings to multiple reference terminologies and connections to several natural languages	combination of OCL, medical logic modules and the vMR subset of HL7 RIM; the HL7 RIM provides a thorough clinical information model; thorough expression possibilities through OCL

**Linkage to standard terminologies**	bindings to any terminology possible through medical logic modules	no intrinsic support, but some platforms provide further functionality. The tool HeCaSe2, for example, supports UMLS.	bindings to terminologies possible through medical concepts	ICD, LOINC	SNOMED CT, LOINC, NDF-RT	bindings to any terminology possible through archetypes and terminology binding section	bindings to any terminology possible through medical logic modules
**Degree of patient data standardisation**	local institution-specific data definitions, leading to the 'curly braces problem'	no standards used	supports HL7	no standards used	HL7 RIM, VMRs derived from HL7 RIM, Clinical Expression Model for data representation consistency	based on openEHR	based on HL7
**Dependence on natural languages**	translation of a guideline representation has to be done manually. The linkage to standard terminologies may facilitate automatic translations, since some terminologies	translation of a guideline representation has to be done manually	translation of a guideline representation has to be done manually. The linkage to standard terminologies may facilitate automatic translations, since some terminologies like	translation of a guideline representation has to be done manually. The linkage to standard terminologies may facilitate automatic translations, since some terminologies	translation of a guideline representation has to be done manually. The linkage to standard terminologies may facilitate automatic translations, since some terminologies	natural-language independency through archetypes, indirect reference to data elements in the rules, language codes indexed meta-data and term_definitions	translation of a guideline representation has to be done manually. The linkage to standard terminologies may facilitate automatic translations, since some terminologies

	like SNOMED CT are available in different natural languages.		SNOMED CT are available in different natural languages.	like SNOMED CT are available in different natural languages.	like SNOMED CT are available in different natural languages.		like SNOMED CT are available in different natural languages.
**Ongoing development and research**	no current further development or research	ongoing research into new applications	ongoing research into new applications	ongoing research into new applications	ongoing research into new applications	ongoing development of the language and research into new applications	ongoing development of the language and research into new applications

HL7 RIM: Health Level Seven Reference Information Model

HL7: Health Level Seven

ICD: International Classification of Diseases

LOINC: Logical Observation Identifiers Names and Codes

NDF-RT: National Drug File-Reference Terminology

OCL: Object Constraint Language

openEHR RM: openEHR Reference information Model

SNOMED CT: Systematised Nomenclature of Medicine Clinical Terms

UMLS: Unified Medical Language System

VMR: virtual medical record
